# Cognitive adaptations for memory deficits in MCI and AD patients: A meta-analysis of EEG microstates

**DOI:** 10.1016/j.nicl.2025.103929

**Published:** 2025-12-24

**Authors:** Timothy Piton, Una Smailovic, Vesna Jelic, Thomas Koenig, Paul G. Unschuld, Lucie Bréchet

**Affiliations:** a Department of Fundamental Neurosciences, University of Geneva, Switzerland; b Division of Clinical Geriatrics, Department of Neurobiology, Karolinska Institute, Sweden; c Department of Clinical Neurophysiology, Karolinska University Hospital, Stockholm, Sweden; d University Hospital of Psychiatry and Psychotherapy, University of Bern, Switzerland; e Geriatric Psychiatry Service, University Hospitals of Geneva (HUG), Thônex, Switzerland; f Department of Psychiatry, University of Geneva, Switzerland; g Department of Clinical Neurosciences, University of Geneva, Switzerland

**Keywords:** Cognitive decline, MCI patients, AD patients, Healthy aging, Resting-state EEG microstates, Meta-analysis, Early diagnosis, Functional biomarker

## Abstract

**Aim:**

EEG microstate analysis enables the exploration of the brain’s neuronal network activity associated with distinct mental states and cognitive functions in neurodegenerative conditions, such as Alzheimer’s disease (AD). Some studies indicated that AD and patients with mild cognitive impairments (MCI) show a reduced presence of microstate C, which is related to self-related memory functions and mind-wandering and involves brain areas of the default mode network (DMN). However, other studies reported an increased presence of microstate A, which is associated with auditory/language functions. Here, we aimed to systematically investigate alterations in the four canonical EEG microstates (A, B, C, and D) in MCI and AD patients compared to healthy older adults.

**Methods:**

We performed a meta-analysis that compared microstate temporal parameters − mean duration, occurrence rate, and time coverage − among MCI and AD patients and healthy older adults. We included 12 experimental studies that examined resting-state, eyes-closed EEG microstate parameters in 1347 participants (448 MCI patients, 514 AD patients, and 385 healthy controls).

**Results:**

We found that AD patients showed increased duration and time coverage of microstate A and increased duration of microstate B, while the occurrence of microstates C and D was reduced. MCI patients also exhibited increased duration, occurrence, and time coverage of microstate A, while all three parameters of microstate D were reduced compared to healthy controls.

**Conclusion:**

These results suggest that MCI and AD patients suffer from disruption in their cognitive control, memory, and self-referential processes. They may compensate for these deficits by verbalizing and visualizing their inner thoughts to maintain cognitive engagement.

## Highlights

• Meta-analysis of EEG microstates in MCI and AD patients versus healthy controls.
• MCI and AD patients show increased microstate A reflecting auditory/language activity.
• Decreased microstate D in MCI and reduced microstates C/D in AD patients.
• Findings indicate a disrupted default mode network and cognitive control processes.
• Patients may compensate for memory deficits by verbalizing and visualizing thoughts.

## 1 Introduction

Alzheimer's disease (AD) becomes increasingly prevalent as the world's population continues to age. Despite its devastating impact, this neurodegenerative brain disease remains incurable (Kim et al., 2022). AD is particularly characterized by progressive cognitive decline and memory loss. AD changes include the pathological accumulation of amyloid-beta plaques, tau neurofibrillary tangles, and neurodegeneration, including brain atrophy and network dysfunction (Serrano-Pozo et al., 2011). As AD progresses, these pathological changes extend from the hippocampus to anatomically connected regions within the default mode network (DMN) (Puttaert et al., 2020). The DMN is one of the large-scale resting-state networks (RSNs) that has been associated with cognitive processes that are directed towards the self, including autobiographical memory and self-referential thoughts, that become disrupted in AD (Buckner et al., 2008, Raichle, 2015). Anatomically, DMN involves a set of brain regions, including the posterior cingulate cortex (PCC), precuneus (PCu), medial prefrontal cortex (MPFC), temporoparietal junction (TPJ), and hippocampus, that become active during spontaneous self-related processes at rest (Andrews-Hanna, 2012, Raichle et al., 2001, Spreng and Grady, 2010). These DMN regions overlap with brain areas with high levels of amyloid-beta and tau protein deposition, brain atrophy, and hypometabolism in AD, which suggests that the DMN is particularly vulnerable to AD pathology (Buckner et al., 2008, Buckner et al., 2005).

The pathological changes begin years before the onset of the symptoms in AD. Evidence suggests that synaptic loss and brain functional deficits occur early in AD and are closely linked to clinical symptoms (John & Reddy, 2021). The early detection of AD is thus crucial for postponing the disease progression. As such, focusing on the early stages of these clinicopathological processes, known as mild cognitive impairment (MCI), seems important (Puttaert et al., 2020). MCI is characterized by noticeable cognitive deficits greater than expected for an individual's age, yet these deficits do not significantly interfere with daily functioning. Cognitive impairment in subjects with MCI is not attributable to normal aging and may be progressive or dissolve depending on possible treatment or underlying causes (WHO report, 2021). Identifying the neurophysiological correlates of MCI cognitive deficits may potentially serve as a target for early intervention to prevent or delay the onset of dementia. This motivates the pursuit of novel biomarkers of neuronal dysfunction in patients along the AD continuum.

Human brain activity is intrinsically organized into RSNs that transiently activate or deactivate at sub-second timescales (Bressler and Tognoli, 2006, Rubega et al., 2019). However, most neuroimaging studies apply resting-state functional connectivity magnetic resonance imaging (rs-fcMRI), which cannot address how AD affects these fast-temporal brain dynamics. Electroencephalography (EEG) is a noninvasive neurophysiological technique that reflects this sub-second real-time neuronal activity dynamics (Michel et al., 2009). EEG captures the spontaneous oscillatory patterns of neuronal populations, providing valuable insights into whole-brain neuronal synchronization and its dysfunction, such as in AD pathology (Koenig et al., 2005, Lopes da Silva, 2013).

One intriguing phenomenon observed in EEG data is the presence of EEG microstates, brief, quasi-stable topographical configurations of neural activity lasting tens to hundreds of milliseconds (Koukkou and Lehmann, 1987, Michel and Koenig, 2018). The time course of these microstates represents the temporal organization of the intrinsic large-scale neuronal networks of the brain (Koenig et al., 2005). It has been shown that a few microstate topographies dominate in resting-state EEG recordings. Four microstates, conventionally labelled A, B, C, and D, have received particular attention in the literature, even though more microstates have been repeatedly described in recent studies (Michel et al., 2024). Combined EEG-fMRI (Britz et al., 2010, Van de Ville et al., 2010) and EEG source imaging studies imaging (Custo et al., 2017) have shown that these microstates are closely linked to the large-scale RSN identified by fMRI. In the context of this paper, it is important to mention the association of microstate C with the self-directed inner processes that underlie the DMN (Bréchet et al., 2019, Koenig et al., 2024). In general, alterations in EEG microstate temporal dynamics are sensitive to disturbances in large-scale RSNs in different neuropsychiatric disorders, e.g., schizophrenia, depression, bipolar disorders, or AD (Chivu et al., 2023, Khanna et al., 2015, Koenig et al., 2020). As such, EEG microstates may offer a unique window into understanding the underlying neurophysiological alterations associated with AD progression.

Several studies have demonstrated association of alterations in EEG microstate parameters with specific clinical features of AD, including disease severity, cognitive decline, and conversion from MCI to AD. Smailovic et al. (2019) recently investigated differences in EEG microstate topographies and temporal parameters in 210 subjective cognitive decline (SCD) subjects, 230 MCI, and 197 AD patients. This study showed a gradient-like increase in microstates A and B, while microstates C and D decreased with more severe stages of cognitive impairment. Similarly, Musaeus et al. (2020) showed that 117 MCI and 117 AD patients had significantly increased temporal parameters of microstate A compared to 135 healthy controls. These studies indicate that cognitive impairment seems to be characterized by the increased presence of microstates A and B and a decrease in microstates C and D. However, other studies did not report differences in microstates between MCI/AD patients and healthy controls (Grieder et al., 2016, Lian et al., 2021, Nishida et al., 2013, Schumacher, et al., 2020).

Here, we aimed to synthesize existing evidence from previous studies on EEG microstates in MCI and AD patients compared to healthy controls. We performed a meta-analysis of three EEG microstate temporal parameters: duration, occurrence, and coverage. We examined whether microstate alterations are consistently associated with cognitive impairment, potentially distinguishing between MCI and AD patients compared to healthy controls. Further, we aimed to identify specific microstate features that are most sensitive for detecting cognitive decline due to AD, possibly aiding in developing diagnostic biomarkers for early detection and intervention of AD. By gathering prior studies using the EEG microstate approach and identifying patterns of EEG temporal alterations in MCI and AD patients, we hope this meta-analysis can further advance our understanding of cognitive decline and aberrant EEG microstate dynamics in MCI and AD patients.

## 2 Methods

### 2.1 Search and selection

We systematically searched the literature to identify experimental studies that provided temporal parameters of resting-state EEG microstates in MCI patients, AD patients, and healthy older adults. We used the following keywords in the search: (i) “EEG microstates” and “mild cognitive impairment” and (ii) “EEG microstates” and “Alzheimer's disease” to search the three databases: PubMed, PsychInfo, and Web of Science. As the output given by the last two databases appeared redundant to that provided by PubMed, we selected the PubMed search results. The publication date of the results ranged from April 1997 to September 2024. We further screened the articles from the systematic search for potential inclusion in the meta-analysis. Articles were excluded if they did not specifically target the examination of temporal parameters in either MCI or AD patients if they were a review article or an opinion paper.

### 2.2 Eligibility criteria

After screening the experimental studies on EEG microstates in MCI and AD patients vs. healthy adults, we included studies that reported the descriptive statistics (i.e., mean (*μ*), and standard deviation (*SD*) of the duration, occurrence rate, and coverage of at least the four canonical microstates generally labeled in the literature as A, B, C, and D (Koenig et al., 2002). Eligible studies had to provide microstate temporal parameters derived from topographic clustering of EEG signals recorded during resting state periods with eyes closed. The included studies had to comprise either i.) clinically diagnosed MCI patients and age-matched healthy adults or ii.) clinically diagnosed AD patients and aged-matched healthy adults. Studies without age-matched controls were excluded from the meta-analysis. Notably, studies investigating AD patients with comorbidities (e.g., Down syndrome) or studies that reported microstate temporal parameters only after interventions (e.g., pharmacological or brain stimulation) were excluded from subsequent analysis. The studies included in this meta-analysis were performed in accordance with the Declaration of Helsinki and approved by the Institutional Ethical Review Board or the Regional Ethics Committee. All participants signed an approved informed consent form.

### 2.3 Data extraction

Data extracted from the experimental studies in this meta-analysis included: (1) study authors; (2) publication year; (3) sample demographics (number of participants, mean age, % female participants, mean Mini-Mental State Examination (MMSE) or Montreal Cognitive Assessment (MoCA) score), (4) total EEG recording duration; (5) number of electrodes used; (6) number of epochs analyzed; (7) epoch duration; (8) pre-processed EEG data duration; (9) number of microstate topographies identified; (10) segmentation algorithm used; (11) microstate segmentation software used; (12) descriptive statistics (i.e. *μ*, *SD*) of each microstate's duration, occurrence rate and coverage.

### 2.4 Microstate temporal parameters

The microstate temporal parameters were extracted and grouped according to each microstate configuration (i.e., A, B, C, D) and each population (i.e., MCI patients, AD patients, and healthy older adults). This classification was done according to the labeling provided by the authors, which we then visually compared with the standardized configurations (Koenig et al., 2024, Koenig et al., 2002). We focused on the descriptive statistics (*μ* and *SD*) of three microstate temporal parameters: duration, occurrence rate, and time coverage. The duration of a microstate in a given individual corresponds to the mean of the durations of each segment of the analyzed EEG epochs identified as this microstate. The occurrence rate of a microstate is defined as the average rate per second at which this microstate occurs. The time coverage of a microstate refers to the percentage of the analyzed EEG time series this microstate occupies. Due to the scarcity of studies providing these values, we excluded other microstate parameters, such as transition probabilities and global explained variance.

### 2.5 Statistical analysis

We computed the standardized mean difference (SMD) of the three temporal parameters between i.) the MCI patients and the age-matched healthy older adults and ii.) the AD patients and the age-matched healthy older adults. We used the *Hedge's g* as effect size. The overall weighted effect size was then calculated across the studies using a two-level mixed effects model, and the implementation provided by the *meta* R package was based on the *metafor* implementation (Balduzzi et al., 2019). The reported effects were weighted according to the inverse variance method. We provide the 95 % confidence interval and the t-distribution-based prediction interval of the mixed models' effect size. The *α-*threshold, determining our significance level, was set to 0.05.

## 3 Results

### 3.1 Systematic search

**Fig. 1:**
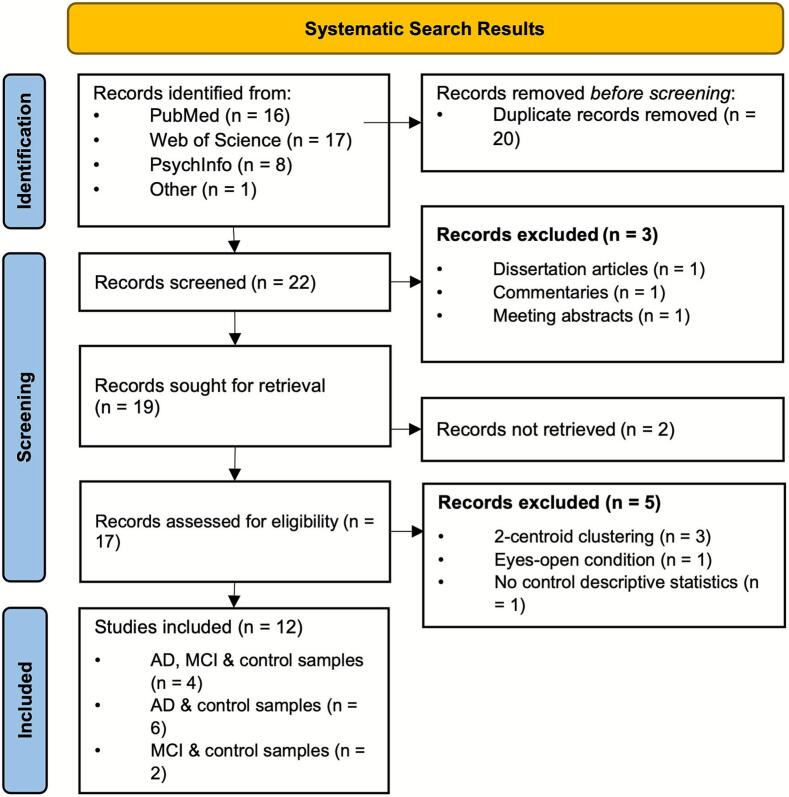
A flow diagram of the systematic search and selection process was conducted for this meta-analysis comparing temporal parameters (duration, time coverage, occurrence) of resting-state EEG microstates in MCI patients, AD patients, and age-matched healthy older adults.

We identified a total of 42 studies from the search of online databases. Nineteen relevant publications were further investigated for eligibility. Twelve studies met all the eligibility criteria and reported sufficient information to be included in the meta-analysis. All included studies were published after 2010, between 2013 and 2024, with a median publication year of 2021. A summary of the systematic search is provided in Fig. 1. Six studies (Lassi et al., 2023, Li et al., 2024, Lian et al., 2021, Musaeus et al., 2020, Musaeus et al., 2019, Tarailis et al., 2025) included in this meta-analysis compared MCI patients (*k* = 293) to healthy older adults (*k* = 324), including a total of 617 participants. For a summary of studies comparing MCI patients with healthy controls, see Table 1.

**Table 1:** Summary of studies included in the meta-analysis comparing MCI patients and HC. The group-level EEG microstate maps were presented separately for MCI patients and HC or together in each publication.

**MCI vs. HC**									
**Authors**	**Participants**	**Age (years)**	**Gender**	**MMSE**	**Microstate maps**				**Main findings**
[1] Musaeus et al. (2019)	25 MCI 37 HC	71.4 (6.0)65.7 (6.9)	6 female 17 female	27.6 (1.5)29.1 (1.0)					↑ occurrence & coverage in MCI of microstate A
[2] Musaeus et al. (2020)	117 MCI 135 HC	70.15 (8.13)66.44 (7.64)	53 % female 60.7 % female	27.11(2.16) 28.91 (1.34)					↑ duration, occurrence & coverage in MCI of microstate A
[3] Lian et al. (2021)	46 MCI 43 HC	71.4 (7.1)70.7 (6.1)	22 female 18 female	24.3 (3.3)27.5 (1.5)					no statistical differences in temporal parameters between MCI and HC
[4] Lassi et al. (2023)	46 MCI 19 HC	74.26 (8.66)64.29 (4.77)	29 female 8 female	26.82 (2.23)29.06 (1.13)					↓ duration & coverage of microstate C in MCI
[5] Li et al. (2024)	29 MCI 30 HC	65.96 (4.35)64.64 (3.18)	20 female 16 female	18.9 (2.35) −					↑ duration, occurrence & coverage in MCI of microstate B
			
[6] Tarailis et al., 2025	30 MCI 60 HC	71.13 (7.92)71.27 (7.92)	13 female 34 female	23.4 (4.15)26.73 (2.56)					↑ duration, occurrence & coverage in MCI of microstate A
			

Abbreviations: MCI = mild cognitive impairment; HC = healthy controls; MMSE = Mini-Mental State Exam; values are in mean (SD).

Ten studies (Grieder et al., 2016, Hanoglu et al., 2022, Li et al., 2024, Lian et al., 2021, Musaeus et al., 2020, Musaeus et al., 2019, Nishida et al., 2013, Schumacher, et al., 2020; Teipel et al., 2021, Yan, et al., 2024) comprising a total of 701 participants compared AD patients (*k* = 346) to healthy older adults (*k* = 355). One of the included studies (Nishida et al., 2013) did not report the descriptive statistics of time coverage. Therefore, nine studies were included for the time coverage differences between AD patients (*k* = 327) and healthy controls (*k* = 336). For a summary of studies comparing AD patients with healthy controls, see Table 2.

**Table 2:** Summary of studies included in the meta-analysis comparing AD patients and HC. The group-level EEG microstate maps were either presented separately for AD patients and HC or together in each publication.

**AD vs. HC**									
**Authors**	**Participants**	**Age (years)**	**Gender**	**MMSE**	**Microstate maps**				**Main findings**
[1] Nishida et al. (2013)	19 AD	68.28 (7.96)	13 female	21.05 (2.44)					no statistical differences in temporal parameters between AD and HC
	19 HC	65.68 (6.10)	11 female	28.74 (1.91)					no statistical differences in temporal parameters between AD and HC
[2] Grieder et al. (2016)	8 AD	65.1 (5.6)	−	22.4 (3.9)					
	8 HC	66.0 (4.6)		28.5 (1.9)					↑ occurrence & coverage in AD of microstate A
[3] Musaeus et al. (2019)	15 AD	70.1 (7.8)	8 female	26.3 (3.2)					
	37 HC	65.7 (6.9)	17 female	29.1 (1.0)					
[4] Schumacher et al. (2019)	27 AD	74.9 (7.0)	7 female	20.7 (4.3)					no statistical differences in temporal parameters between AD and HC
	18 HC	76.3 (5.5)	7 female	29.2 (0.9)					
[5] Musaeus et al. (2020)	117 CE	75.49 (7.65)	60.7 %	23.52 (3.79)					↑ duration, occurrence & coverage in AD of microstate A
	135 HC	66.44 (7.64)	60.7 %	28.91 (1.34)					
[6] Teipel et al. (2021)	14 AD	75.3 (5.7)	4 female	24.6 (3.1)					↑ occurrence of microstate B & ↓ occurrence of microstate C in AD
	14 HC	73.4 (3.1)	4 female	28.7 (0.8)					
[7] Lian et al. (2021)	43 AD	73.8 (7.0)	23 female	18.6 (6.6)					↑ duration & coverage in microstate B in AD; ↓ coverage of microstate C in AD
[8] Hanoglu et al. (2022)	18 AD	70.67 (7.71)	12 female	17.93 (4.46)					↑ occurrence & coverage in AD of microstate A; ↑ duration, occurrence & coverage in AD of microstate B; ↓ duration & coverage of microstates C and D in AD
	13 HC	69.15 (5.60)	5 female	27.46 (1.33)					
[9] Li et al. (2024)	29 AD	69.82 (9.32)	11 female	11.48 (5.50)					↑ duration in AD of microstates C & D; ↓ occurrence of microstate B
	30 HC	64.64 (3.18)	16 female	–					
[10] Yan et al. (2024)	56 AD	62.22 (8.409)	31 female	18.72 (6.364)					no statistical differences in temporal parameters between AD and HC
	38 HC	60.28 (7.096)	24 female	28.46 (1.401)					

Abbreviations: AD = Alzheimer's disease; HC = healthy controls; MMSE = Mini-Mental State Exam; values are in mean (SD).

### 3.2 Participants

In total, 1073 participants were included in this meta-analysis (including 293 MCI patients, 346 AD patients, and 434 healthy older adults). The mean age of the MCI patients was 70.5 ± 2.2 years. The mean age of the AD patients was 71.2 ± 4.8 years. The mean age of the healthy adults was 67.4 ± 3.7 years. On average, the studies comprised 45.0 ± 10.0 % male individuals among the AD patients, 48 ± 10 % among the MCI patients, and 45.4 ± 8.8 % among healthy older adults.

### 3.3 The Mini-Mental State Examination (MMSE)

The Mini-Mental State Examination (MMSE) (Folstein et al., 1975) and the Montreal Cognitive Assessment (MoCA) (Nasreddine et al., 2005) are widely used cognitive screening tools for assessing cognitive function and detecting impairments (Wang et al., 2022). The mean MMSE score was 26.4 ± 1.3 for the MCI patients, 21.2 ± 2.6 for the AD patients, and 28.7 ± 0.5 for healthy older adults. Two studies (Li et al., 2024, Tarailis et al., 2025) reported the Montreal Cognitive Assessment (MoCA) score, while others reported the MMSE score. Therefore, we transformed the MoCA scores into equivalent MMSE scores (Fasnacht et al., 2023) and calculated each study's mean MMSE score and standard deviation.

### 3.4 EEG data recordings and pre-processing

The median number of electrodes used for EEG recordings was 31, ranging from 19 to 256. The average raw EEG recording length was approximately 9 min, with recordings as short as 2.5 min and as long as 22 min. 8.0 % of the included studies used a high-pass cutoff frequency of 2 Hz and a low-pass cutoff frequency of 20 Hz. The rest employed a wider frequency range, with a high-pass cutoff at 1 Hz and a low-pass cutoff between 20 Hz and 40 Hz. Most (83.3 %) of the studies set the sampling frequency to values ranging from 128 Hz to 500 Hz, with only two studies using higher rates.

### 3.5 Microstates segmentation

Microstate cluster topographies were computed using the modified *k*-means clustering algorithm in 75.0 % of the studies (Pascual-Marqui et al., 1995). Three studies used the AAHC or TAAHC algorithm (Murray et al., 2008). All included studies identified or fixed the optimal number of clusters to four configurations, except one study that identified five clusters using the *meta*-criterion proposed by Michel and Brunet (2019). Of the studies that reported the software used for microstate segmentation and analysis (*k* = 7), six used the EEGLAB Microstate plugin, and one worked with Cartool.

### 3.6 MCI patients vs. healthy controls

• **The duration of microstate A increased while D decreased**

We applied the random effects model estimate that indicated a significantly larger mean duration of microstate A in the MCI patients compared with the healthy controls (*g* = 0.398, 95 % CI = [0.163; 0.632], PI = [0.119; 0.676], *z|t* = 4.35, *p* = 0.007). The data of microstate B revealed a non-significant SMD estimate of *g* = 0.247 (95 % CI = [−0.251, 0.745], PI = [−1.002, 1.496], *z|t* = 1.27, *p* = 0.259). The mean duration of microstate C showed a non-significant SMD of *g* = −0.220 (95 % CI = [−0.668, 0.227], PI = [−1.269, 0.829], *z|t* = −1.27, *p* = 0.261). The SMD estimate of microstate D was significant (*g* = −0.306, 95 % CI = [−0.500, −0.111], *z|t* = −4.04, *p* = 0.010), indicating shorter duration in MCI patients compared to healthy controls.

• **The occurrence rate of microstate A increased while D decreased**

We fitted the random effects model to the occurrence rate data of microstate A and obtained an effect size estimate of *g* = 0.369 (95 % CI = [0.152; 0.586], PI = [0.138; 0.600], *z|t* = 4.37, *p* = 0.007), pointing to significantly higher occurrence rates of microstate A in MCI patients compared to healthy controls. The data of microstate B revealed a non-significant SMD estimate of *g* = 0.165 (95 % CI = [−0.250, 0.579], PI = [−0.804, 1.133], *z|t* = 1.02, *p* = 0.354). The SMD estimate of microstate C was not significant (*g* = −0.180, 95 % CI = [−0.586, 0.226], PI = [−1.145, 0.785], *z|t* = −1.14, *p* = 0.306). We found a significant SMD estimate of microstate D (*g* = −0.190, 95 % CI = [−0.500, −0.111], *z|t* = −4.04, *p* = 0.010), pointing out significantly lower occurrence rates of microstate D in MCI patients compared to those observed in healthy controls.

• **The time coverage of microstate A increased while D decreased**

**Fig. 2:**
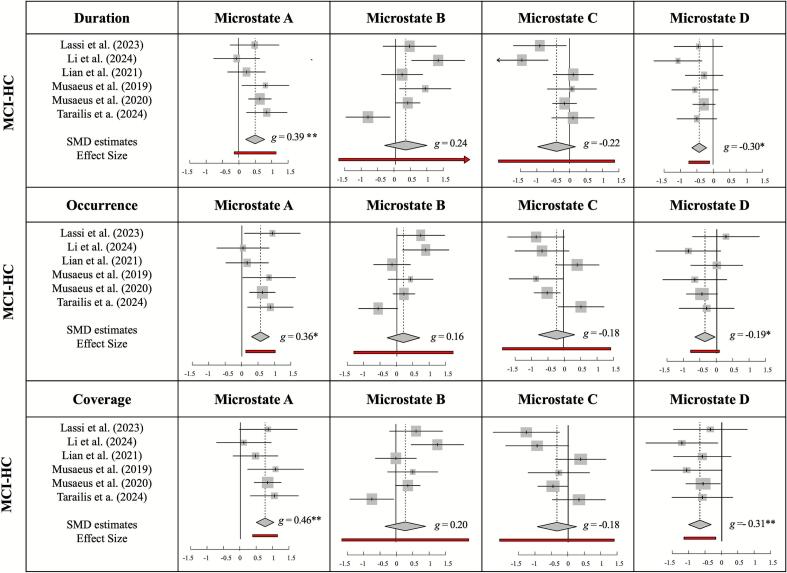
Forest plots of meta-analysis results of mild cognitive impairment patients vs. healthy older adults for each microstate (i.e., A, B, C, D) and temporal parameters (i.e., duration, occurrence, time coverage). Hedges' *g* (squares proportional to weights used in meta-analysis) and associated confidence intervals (lateral tips) for individual data sets; summary measures (diamonds) of the whole group.

We applied the random effects model that we fitted to the coverage data of microstate A and found a significant effect size estimate of *g* = 0.462 (95 % CI = [0.262; 0.662], PI = [0.231; 0.694], *z|t* = 5.95, *p* = 0.002). This result suggests that microstate A covers a larger time period in MCI patients than in healthy older controls. A random effects model fitted to microstate B time coverage revealed a non-significant SMD of *g* = 0.208 (95 % CI = [−0.250, 0.666], PI = [−0.906, 1.322], *z|t* = 1.17, *p* = 0.295). The weighted estimate was not significant for microstate C (*g* = −0.182, 95 % CI = [−0.545, 0.182], PI = [−0.968, 0.605], *z|t* = −1.28, *p* = 0.256). The fitted random effects model's time coverage SMD estimate of microstate D was significant (*g* = −0.319, 95 % CI = [−0.454, −0.184], *z|t* = −6.07, *p* = 0.002), indicating that the time coverage of microstate D was significantly lower in MCI patients compared to healthy controls. The summary of the results is depicted in Fig. 2.

### 3.7 AD patients vs. healthy controls

• **The duration of microstates A and B increased in AD patients**

Applying a random effects model to microstate A yielded an effect size estimate of *g* = 0.440 (95 % CI = [0.216; 0.663], PI = [−0.040; 0.919], *z|t* = 4.46, *p* = 0.002). This result indicates a significantly longer mean duration of microstate A in AD patients compared to the age-matched healthy controls. The data gathered for microstate B revealed a significant effect size estimate of *g* = 0.399 (95 % CI = [0.262; 0.537], PI = [0.221; 0.578], *z|t* = 6.58, *p* = 0.0001), implying that the mean duration of microstate B is significantly longer in AD patients than in healthy controls. Microstates C yielded a non-significant SMD estimate of *g* = −0.075 (95 % CI = [−0.361, 0.212], PI = [−0.733, 0.584], *z|t* = −0.59, *p* = 0.569). Likewise, microstate D showed a non-significant SMD estimate of *g* = −0.238 (95 % CI = [−0.507, 0.031], PI = [−0.753, 0.277], *z|t* = −2.01, *p* = 0.076).

• **The occurrence rate of microstates C and D decreased in AD patients**

The random effects model of microstate A did not provide a significant SMD estimate (*g* = 0.204, 95 % CI = [−0.067; 0.476], PI = [−0.490; 0.898], *z|t* = 1.70, *p* = 0.123). Likewise, microstate B showed a non-significant effect size estimate of *g* = 0.158 (95 % CI = [−0.271; 0.586], PI = [−0.981; 1.296], *z|t* = 0.83, *p* = 0.426). A significant SMD estimate was found for microstate C (*g* = −0.213 (95 % CI = [−0.389, −0.036], PI = [−0.391, −0.036], *z|t* = −2.76, *p* = 0.022), suggesting that the occurrence rate of microstate C is significantly lower in AD patients than that of healthy older controls. Similarly, a random effects model applied to the data of microstate D showed a significant SMD estimate of *g* = −0.304 (95 % CI = [−0.457, −0.151], PI = [−0.523, −0.085], *z|t* = −4.50, *p* = 0.002), which implies that microstate D occurs significantly less often in AD patients than in healthy controls.

• **The time coverage of microstate A increased in AD patients**

**Fig. 3:**
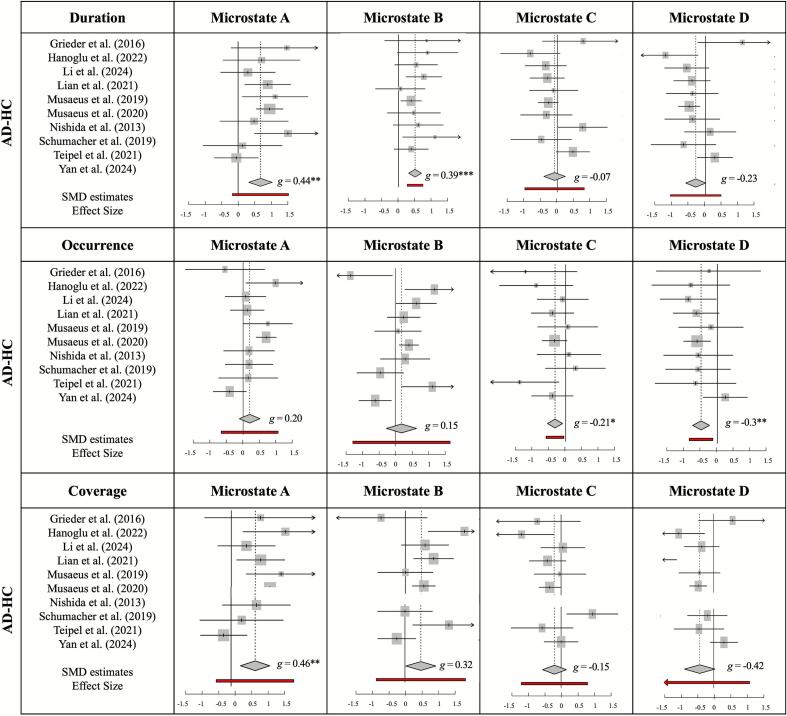
Forest plots of meta-analysis results of Alzheimer's patients vs. healthy older adults for each microstate (i.e., A, B, C, D) and temporal parameters (i.e., duration, occurrence, time coverage). Hedges' *g* (squares proportional to weights used in meta-analysis) and associated confidence intervals (lateral tips) for individual data sets; summary measures (diamonds) of the whole group.

We fitted the random effects model to microstate A and found a significantly larger time coverage of microstate A in AD patients compared to healthy controls (*g* = 0.468, 95 % CI = [0.198; 0.738], PI = [−0.198; 1.135], *z|t* = 4.00, *p* = 0.004). The effect size estimate of microstate B was not significant (*g* = 0.328, 95 % CI = [−0.058, 0.714], PI = [−0.585, 1.241], *z|t* = 1.96, *p* = 0.086). The weighted SMD estimate provided by the fitted random effects model of microstate C was not significant (*g* = −0.151, 95 % CI = [−0.473, 0.172], PI = [−0.844, 0.543], *z|t* = −1.08, *p* = 0.313). The effect size yielded by the random effects model fitted to the data of microstate D was not significant (*g* = −0.425, 95 % CI = [−0.905, 0.056], PI = [−1.798, 0.949], *z|t* = −2.04, *p* = 0.076). A summary of the results is depicted in Fig. 3.

## 4 Discussion

In this study, we aimed to provide insights into the EEG microstate temporal alterations in MCI and AD patients compared to healthy older adults. The main findings of our meta-analytical investigation showed small yet significant effect sizes. Specifically, we found that the duration and coverage of microstate A and the duration of microstate B increased in AD patients compared to healthy older controls. In MCI patients, all temporal parameters (i.e., duration, occurrence, and coverage) of microstate A increased compared to healthy older controls. On the other hand, the occurrence of microstates C and D decreased in AD patients compared to healthy older controls. In MCI patients, microstate D's duration, occurrence, and coverage decreased compared to healthy older controls.

Previous neuroimaging studies demonstrated that significant alterations occur in the large-scale RSNs of MCI and AD patients (Dillen et al., 2017, Putcha et al., 2022, Wang et al., 2013). These networks are collections of brain regions that show synchronous activity at rest, and their disruption is closely linked to cognitive decline. Emerging evidence suggests that the temporal alterations of EEG microstates reflect disturbances in large-scale RSN dynamics (Michel et al., 2024, Michel and Koenig, 2018). EEG microstates are thus critically related to many cognitive functions, such as autobiographical memory or executive functioning, that are affected in MCI and AD patients (Bréchet et al., 2019, Tarailis et al., 2025). The progression of EEG microstate alterations from the early stages of MCI to the possible later stages of AD dementia has been investigated by multiple studies (Lassi et al., 2023, Lian et al., 2021, Musaeus et al., 2019, Shi et al., 2022), offering valuable insights into the evolution of functional brain network disruptions throughout the disease. Several studies have highlighted differences in microstate dynamics between MCI (Lassi et al., 2023, Li et al., 2024, Musaeus et al., 2020, Musaeus et al., 2019, Tarailis et al., 2025) and/or AD patients (Hanoglu et al., 2022, Li et al., 2024, Lian et al., 2021, Musaeus et al., 2020, Musaeus et al., 2019; Teipel et al., 2021) compared to healthy adults.

While some studies reported increased values for microstates A and B parameters and decreased values for microstates C and D in MCI and AD patients (Lassi et al., 2023, Li et al., 2024, Musaeus et al., 2020, Musaeus et al., 2019, Tarailis et al., 2025; Teipel et al., 2021), others showed no significant differences between patients and healthy controls (Grieder et al., 2016, Nishida et al., 2013, Schumacher et al., 2019, Yan, et al., 2024). In this study, we systematically performed a meta-analysis focusing on microstate temporal parameters − duration, coverage, and occurrence − across healthy and pathologically aging populations. By synthesizing existing evidence from individual studies with MCI and/or AD patients, we aimed to establish conclusions regarding the differences in EEG microstate parameters in these patient populations compared to healthy controls.

Studies using simultaneous EEG-fMRI (Britz et al., 2010, Van de Ville et al., 2010) and EEG source imaging (Custo et al., 2017) have shown similarities between EEG microstates and the large-scale RSN identified by fMRI A systematic review of Tarailis et al. (2023) attributed EEG microstates to specific cognitive functions according to their modulations by task instructions, subjective resting-state questionnaires, and source localization. The authors found that microstates A and B were predominantly related to sensory categories, with microstate A more related to auditory/language functions and microstate B to visual functions. Microstate C was mainly associated with self-oriented and memory functions and microstate D with cognition and attention. Simultaneous EEG-fMRI (Britz et al., 2010) and EEG source imaging (Bréchet et al., 2019, Bréchet et al., 2020, Custo et al., 2017) also attributed microstate A to the auditory/language resting-state network, microstate B to the visual network, microstate C to the default mode or saliency network, and microstate D to the attention network.

Our systematic meta-analysis revealed that the temporal dynamics of microstate C were diminished in AD and to a lower extent in MCI patients compared to healthy older controls. Microstate C has been previously associated with *“the self”*, including *“autobiographical memory”* and *“self-referential thoughts”*. In our recent study (Tarailis et al., 2025), we investigated possible alterations of the spontaneous mentation in 30 MCI patients using a 5-min resting-state hdEEG paradigm with the standard instructions: *“Close your eyes and let your mind wander”*. To examine the inner experiences of participants during rest, we applied the Amsterdam Resting-State Questionnaire (ARSQ). Interestingly, we found diminished self-related thoughts related to personal past experiences (i.e., “I thought about myself,” “I thought about my behavior,” and “I thought about my feelings”), future thinking (i.e., “I thought about the future,” “I thought about things I need to do,” “I thought about solving problems”) as well as visual thoughts (i.e., “I pictured places,” “I pictured events,” “I thought in images”) among the MCI patients compared to healthy younger, but not healthy older participants. These previous findings fit well with our EEG meta-analysis as MCI and AD patients are generally characterized by cognitive decline and memory loss.

Furthermore, using EEG source imaging, Tarailis et al. (2025) showed that microstate C was attributed to activity in the hippocampus, the parahippocampal gyrus, the inferior temporal lobe, the primary visual cortex, the precuneus, and the left angular gyrus. The sources underlying microstate D were localized in the inferior temporal gyrus, the parahippocampal gyrus, the lingual gyrus, and the middle frontal gyrus. It is important to note that while EEG source imaging studies have suggested possible involvement of deep structures such as the hippocampus, these results should be interpreted with caution, as EEG/MEG source localization of resting-state activity from deep structures is limited by low signal-to-noise ratio and increased localization error (Afnan et al., 2024; Bréchet et al., 2020; Pascarella et al., 2023). Therefore, the associations with cortical sources (e.g., precuneus, angular gyrus, temporal cortex) are more reliably established than those with deeper subcortical structures. Our present meta-analytical EEG results, showing diminished values of microstates C and D temporal parameters, correspond well with the neuroimaging findings that show that pathological changes extend from the hippocampus to anatomically connected regions within the DMN as the AD progresses. Interestingly, fMRI studies show that age-related changes in the DMN include a decreased within-network and increased between-network connectivity (Damoiseaux, 2017, Dillen et al., 2017, Spreng and Turner, 2019).

Furthermore, we found here an increase in the temporal parameters of microstate A in MCI and microstate A and B in AD patients that points to increased use of *“thought verbalization”* and *“visual imagery”* during rest in the MCI and AD patients compared to healthy older controls. Our recent experimental study (Tarailis et al., 2025) confirms the present meta-analytical results of a gradient-like increase in microstate A in MCI patients compared to healthy young and older participants. In that study, the hdEEG source localization of microstate A was found in the left inferior and middle temporal gyrus, which are well-known from the neuroimaging literature to subserve language, semantic memory processes, and multimodal sensory integration (Murphy et al., 2023, Onitsuka et al., 2004). Interestingly, cognitive aging is often described in the context of loss and decline. However, emerging research suggests that the story is more complex, with healthy older adults exhibiting cognitive losses and gains. With increasing age, cognition becomes semanticized, meaning goal-directed cognition becomes less dependent on declining control resources and increasingly influenced by prior knowledge (Li-Chay-Chung et al., 2024, Spreng and Turner, 2019). The most commonly reported patterns of functional brain change in older adulthood include greater recruitment of the prefrontal brain regions implicated in cognitive control and reduced suppression of the DMN, a brain network related to self-referential internal thinking (Andrews-Hanna et al., 2014).

In the light of our current meta-analytical results of EEG microstates in MCI and AD patients that show increased temporal parameters, particularly of microstates A and B, which have been previously attributed to auditory/language and visual functions, we here suggest that these individuals may exhibit increased verbalization and mental imagery of self-related thoughts (Milz et al., 2016, Seitzman et al., 2017). On the other hand, our meta-analysis revealed that MCI and AD patients exhibit decreased parameters of microstates C and D, previously linked with DMN, autobiographical memory, and attention (Bréchet et al., 2019, Tarailis et al., 2023). We thus speculate that as MCI and AD patients suffer from disruption in their cognitive control, memory, and self-referential processes, they may compensate for these deficits by verbalizing and visualizing their inner thoughts in an attempt to maintain cognitive engagement. For example, to counteract the cognitive decline, patients may use verbalization, such as: *“After lunch, I need to go upstairs to get my glasses, then I need to put the dishes into the dishwasher.”*

Additionally, MCI and AD patients often suffer from impaired executive control functions, which makes it particularly difficult to suppress their verbal thoughts or internal dialogues (Baudic et al., 2006, Migliaccio et al., 2020). This difficulty of verbal thought suppression may lead even further to visual hallucinations and delusions that may signal cognitive decline and dysfunction in dementia patients (Fuller et al., 2020).

Our initial aim was to identify specific microstate features that may be most sensitive to detecting cognitive decline associated with AD, with the potential to serve as early diagnostic biomarkers. While our findings revealed consistent alterations in specific microstate parameters across MCI and AD groups, particularly the increased temporal parameters of microstate A and the decreased parameters of microstate D, the current evidence does not yet allow their direct application as reliable diagnostic biomarkers. The small to medium effect sizes and methodological heterogeneity across studies indicate that these findings should be viewed as an essential step toward identifying candidate microstate features that warrant further investigation. Future studies employing standardized methodologies, larger samples, and longitudinal designs will be necessary to validate the diagnostic and prognostic value of these microstate alterations and to determine whether they can reliably distinguish between healthy aging, MCI, and AD progression.

**Limitations and future direction**

Our meta-analytical results from individual studies reveal small to medium-sized effects of the EEG microstate temporal properties, which reflect changes in large-scale RSNs in MCI and AD patients. A significant limitation of our investigation is the lack of questionnaires that would elucidate the internal thoughts of participants during rest and the localization of sources of the electrical brain microstates across the individual studies included in our meta-analysis. A further difficulty is the heterogeneity of clinical, demographic, and methodological factors of individual publications included in this meta-analysis.

Another potential concern is whether differences in electrode density across studies (ranging from 19 to 256 electrodes in our meta-analysis) might introduce bias. However, empirical evidence suggests that microstate parameters remain stable across different electrode densities above approximately 20 channels, provided the electrodes are appropriately distributed. Zhang et al. (2021) found few differences in microstate parameters among 91-, 64-, and 32-channel configurations, with high topographic correlations (>0.85–0.95) between densities. Khanna et al. (2014) and Kleinert et al. (2024) similarly confirmed the reliability of microstate identification across varying electrode densities. Given that all included studies employed at least 19 appropriately distributed electrodes, electrode density is unlikely to have introduced systematic bias in our findings.

Another methodological consideration is the variability in EEG recording lengths across studies (2.5 to 22 min). However, microstate parameters show high temporal stability once a minimum threshold of approximately 2–3 min is exceeded. Kleinert et al. (2024) confirmed robust microstate reliability across recording lengths of 2 versus 3 min in over 500 participants, while Van de Ville et al. (2010) demonstrated that microstate temporal dynamics exhibit scale-free behavior across temporal scales, indicating that fundamental properties are preserved across different durations. Given that all included studies used recordings exceeding 2.5 min, recording duration is unlikely to have systematically biased our findings.

A further limitation is the methodological heterogeneity across included studies, including variations in filtering parameters, clustering algorithms, and preprocessing procedures. These methodological differences could contribute to between-study variability in effect sizes. However, the consistency in the direction of effects across studies using diverse methodological approaches suggests that our core findings reflect robust neurophysiological patterns rather than purely methodological artifacts. Future research would benefit from standardized analytical protocols and systematic examination of methodological parameters as potential moderators in larger meta-analytic samples.

It is worth noting that microstate D exhibited greater variability in topography across studies compared to other microstate classes. This variability may reflect inconsistencies in labeling conventions, as definitions of canonical microstates can differ depending on the analysis pipeline, clustering algorithms, and study-specific criteria. Such methodological differences may contribute to some heterogeneity in the reported associations. Therefore, results concerning microstate D should be interpreted with appropriate caution, and future work adopting standardized labeling and analysis approaches will be essential to clarify its role in MCI and AD.

It should be noted that our meta-analysis focused exclusively on EEG microstates. MEG microstate studies examining canonical microstate classes and temporal parameters comparable to those in EEG microstate analysis are currently lacking in MCI and AD populations. Future research applying microstate segmentation methods to MEG data may provide complementary insights into the neurophysiological changes associated with cognitive decline.

**Conclusion**

We conducted a meta-analysis to quantify differences in EEG microstate temporal parameters across healthy older adults, MCI, and AD patients to elucidate the role of EEG microstates in patients on the Alzheimer's disease continuum. We confirmed a gradient-like increase in microstates A and B and a gradient-like decrease in microstates C and D. Prompt diagnosis and treatment of MCI can effectively limit the progression of the disease and its conversion to AD once an effective disease-modifying treatment reaches clinical practice. As research in this field continues to advance, EEG microstates hold the potential to facilitate earlier identification of cognitive impairment and guide the development of novel therapeutic interventions targeting synaptic dysfunction. These interventions could improve cognitive outcomes and the quality of life for individuals affected by AD.

## CRediT authorship contribution statement

**Timothy Piton:** Writing – review & editing, Methodology, Formal analysis. **Una Smailovic:** Writing – review & editing, Methodology. **Vesna Jelic:** Writing – review & editing, Methodology. **Thomas Koenig:** Writing – review & editing, Methodology, Conceptualization. **Paul G. Unschuld:** Writing – review & editing, Conceptualization. **Lucie Bréchet:** Writing – original draft, Visualization, Validation, Supervision, Resources, Project administration, Methodology, Investigation, Conceptualization.

## Data Availability

Data will be made available on request.
